# Associations of perinatal characteristics with endometriosis: a nationwide birth cohort study

**DOI:** 10.1093/ije/dyz140

**Published:** 2019-07-03

**Authors:** Menghan Gao, Kirk Scott, Ilona Koupil

**Affiliations:** 1 Department of Public Health Sciences, Karolinska Institutet, Stockholm, Sweden; 2 Centre for Economic Demography & Department of Economic History, Lund University, Lund, Sweden; 3 Department of Public Health Sciences, Stockholm University, Stockholm, Sweden

**Keywords:** Maternal smoking, birthweight, fetal growth, endometriosis, Sweden, total-population cohort

## Abstract

**Background:**

Perinatal characteristics are associated with subsequent risk of several chronic diseases. Previous studies regarding endometriosis were based on small samples and retrospective data and were limited by unmeasured confounding bias, leading to conflicting and inconclusive findings. We investigated the associations of maternal and birth characteristics with risk of endometriosis among Swedish women of reproductive age.

**Methods:**

This total-population register-based cohort study consisted of 628 312 singleton women born in Sweden between 1973 and 1987, who were followed for diagnosed endometriosis from age 15 years until the end of 2012. Multivariable Cox regression was applied to examine associations with perinatal characteristics. Residual unmeasured confounding was assessed through within-family and E-value analyses.

**Results:**

During follow-up, 8262 women received an endometriosis diagnosis. There were clear dose−response/linear associations of endometriosis with lower maternal education, endometriosis in the mother [adjusted hazard ratio (aHR): 2.24, 95% confidence interval (CI): 2.04–2.46], maternal smoking during pregnancy (aHR: 1.18, 95% CI: 1.04–1.33 for moderate smoker and aHR: 1.36, 95% CI: 1.18–1.57 for heavy smoker vs non-smoker), lower birthweight, and lower birthweight-for-gestational age (aHR: 0.93 per standard deviation increase, 95% CI: 0.91–0.95). Within-family and E-value analyses suggested that these perinatal characteristics are robust predictors of the incidence of endometriosis. We also found that an estimated 26% of the association between maternal smoking and early-onset endometriosis could be explained by birthweight-for-gestational age.

**Conclusion:**

This study finds support for fetal origins of endometriosis, in that exposure to adverse environment or restricted development during the perinatal period may increase the risk. Further research is needed to provide an understanding of the underlying mechanisms.

**Key Messages**

- Maternal smoking during pregnancy was associated with an increased incidence of early onset endometriosis in the daughter.
- Women born with lower birthweight and lower birthweight-for-gestational age were at higher risk of endometriosis during early to mid-reproductive period irrespective of their gestational age at birth.
- The association between maternal smoking during pregnancy and elevated risk of endometriosis seems partly mediated through slow fetal growth rate.
- This study finds support for fetal origins of endometriosis, in that exposure to adverse environment or restricted development during the perinatal period may increase the risk, independent of shared maternal-level confounding.

## Introduction

As proposed by the developmental origins of health and disease theory, and supported by many epidemiological studies,^1^ the relationship between adverse environmental conditions in prenatal or postnatal periods and poor health in later life has become well established.^2^^,^^3^ The hypothesis claims that, apart from the predetermined genetic influence, the human body is susceptible to many modifiable factors during early development such as maternal nutrition and intrauterine environment.^4^^,^^5^ The developmental influence is of particular importance to adult hormone-related disorders, as alterations in prenatal endocrine status or hormonal milieu may permanently reprogramme the fetus’s postnatal hormone secretion and tissue-specific hormone susceptibility.^2^ The theory of developmental origins of disease highlights the need to investigate factors occurring during the perinatal period and their underlying processes in contributing to the onset of hormone-driven disease.

Endometriosis is an oestrogen-dependent, chronic gynaecological condition, defined as the presence of endometrial stroma and glands outside their usual physiological site.^6^ The development of endometriosis is affected by variations in oestrogen and progesterone levels, and commonly manifests among premenopausal women.^7^ The estimated incidence peak of endometriosis is around age 30.^8^ Considering such a young age, it has been suggested that the programming of endometriosis could occur as early as the perinatal period of life.^9^ Despite this, studies regarding the risk factors for endometriosis have mainly focused on factors during adulthood (e.g. shorter menstrual cycles, smaller number of live-born children, lower body mass index).^10–12^ Current epidemiological findings with regard to the early life predictors of endometriosis are conflicting and inconclusive. For instance, exposure to smoking *in utero* has been reported as being inversely associated with the risk in one study,^13^ whereas no association was found in other studies.^9^^,^^14^ Findings with respect to the associations of birth characteristics with endometriosis risk were also complex. Some studies suggested that preterm birth or lower birthweight predict increased risk of endometriosis in adulthood,^9^^,^^15–17^ whereas others do not find support for the fetal origins of endometriosis.^14^^,^^18^

The inconsistent evidence may reflect methodological limitations, including small samples or small numbers of cases and controls,^13^^,^^14^^,^^16^ retrospectively self-reported perinatal factors,^9^^,^^14^^,^^18^ and potential misclassification of endometriosis.^9^^,^^18^ Moreover, in order to ensure that the associations between perinatal characteristics and endometriosis are not spurious, appropriate control for family/maternal-level confounding is needed. Family comparison designs offer an opportunity to reduce the unmeasured genetic or environmental influences.

Using prospectively collected longitudinal data representing the total Swedish female population as well as both traditional and family designs, the current study investigates the associations of maternal and birth characteristics with incidence of endometriosis.

## Methods

### Study population

The study uses the Swedish Interdisciplinary Panel (SIP) database, which contains data from several national registers for the entire Swedish population born between 1973 and 1995, as well as their parents born outside the main sampling window.^19^ We used SIP to generate a cohort of singleton women born in Sweden between 1973 and 1987 (index women), and further identified and linked their biological mothers’ and sisters’ (born from same biological mother) information through unique study numbers provided by the Medical Birth Register (MBR). Beginning with 688 412 women, we excluded those who died (0.8%) or emigrated (3.4%) before age 15 (the start of follow-up). We further excluded 5.3% of the women due to missing data on explanatory variables, and 0.007% women due to implausible combinations of birthweight and gestational age, which resulted in a final study population of 628 312 women.

### Ascertainment of endometriosis

According to the *International Classification of Diseases* (ICD), endometriosis was defined as the abnormal presence of endometrial-type tissue at any site, code 617 (*Ninth Revision*, for the years 1987–1996) and code N80 (*Tenth Revision*, for the years 1997–2012). Cases were identified as women with a main or contributory diagnosis from the Swedish National Patient Register (NPR). The NPR covers all public and private hospital discharges from inpatient care and data recorded by public caregivers for outpatient care.^20^ The inpatient register has complete national coverage from 1987, whereas the outpatient register began in 2001 and with a relatively lower coverage (around 80%).^20^ The reported validity of endometriosis diagnoses in the inpatient register is very good with a positive predictive value of 97.8%.^20^

### Explanatory variables

Mother’s highest educational level was measured as the highest achieved education reported in the Education Register during the years 1987–2012, and categorized as elementary education, secondary education, shorter post-secondary <3 years, and university degree ≥3 years. The MBR provided data on mother’s parity (1, 2, 3, ≥4). Mother’s age at childbirth (≤20, 21–34, ≥35 years) and country of origin were obtained from the Total Population Register. Countries were further categorized according to their similarities with respect to socio-cultural context. For mothers born outside Sweden, length of stay since immigration was calculated. Mother’s lifetime endometriosis diagnosis was identified using the NPR as described above. Maternal smoking during pregnancy was prospectively collected at first visit to antenatal care (usually at 8–12 weeks of gestation^21^) for a subgroup of index women who were born between 1982 and 1987, as information on smoking is only available in the MBR since 1982.

The MBR also provided index women’s birth characteristics, including birthweight in kilograms and gestational age in completed weeks. We calculated standardized birthweight-for-gestational age on a week-by-week manner using all female live births between 1973 and 1987 as the reference. We further categorized infants who were in the lower and upper deciles of birthweight-for-gestational age as small- and large-for-gestational age (SGA/LGA), those in between were classified as appropriate-for-gestational age (AGA). Women’s birth county within Sweden was included and classified into three groups based on the Nomenclature of Territorial Units for Statistics 1.^22^

### Statistical analysis

To assess the associations of perinatal characteristics with risk of endometriosis, Cox proportional hazards regression was applied to estimate hazard ratios (HRs) and 95% confidence intervals (95% CIs), with robust standard errors to account for the correlations between sisters born to the same mother. Using age as the underlying timescale, women were followed from when they turned 15 years of age. Follow-up continued until first diagnosis of endometriosis, or until death, emigration, or 31 December 2012, whichever was earliest. The proportional hazards assumption was tested through Schoenfeld residuals method and met for the main explanatory variables. The linear association between birthweight-for-gestational age and incidence of endometriosis was verified by including a quadratic term into the model (*P* value for quadratic term = 0.42).

Associations of explanatory variables with endometriosis were adjusted for birth year of the index women. Next, measured maternal characteristics (except the one examined) were added into the model to account for potential confounding. Since mother’s smoking is only available for a subgroup of women, it was examined separately and restricted to women who have complete data on the confounding variables included in the model.

To further assess whether the observed associations were likely to be a product of residual maternal/familial-level confounding, we conducted within-family comparisons for characteristics that vary between sisters. We generated a ‘between-mother’ variable, which is the average level of the exposure variable of question within each mother, and a ‘within-mother’ variable to present the deviation of each study subject from the family mean (clustered in mother).^23^ These two new variables were then simultaneously included in the Cox models together with women’s birth year. After fitting the models, a Wald test was performed to examine the difference between the ‘within-mother’ and the ‘between-mother’ coefficients. If such difference was significant we concluded that there was shared residual maternal-level confounding on the examined association, and vice versa.^23^ This method was originally proposed by Mann et al.^23^ and has been utilized in studies of the developmental origins of health outcomes later in life.^23^^,^^24^

We performed additional analyses to explore the potential mediation effect of birth characteristics on the associations between maternal characteristics and incidence of endometriosis. We followed the g-computation procedure (gformula in STATA) to assess the total, direct, and indirect associations, whereas standard errors were estimated with bootstrapping.^25^ All measured confounding factors for the exposure, mediator and outcome associations were included in the mediation model. The model allowed exposure−mediator interaction in the presence of such statistically significant evidence. The direct association was estimated at the mean of the mediator in that case.

### Sensitivity analyses

First, to evaluate the robustness of our results with respect to endometriosis diagnoses recorded by different medical caregivers (or severity of the disease), we restricted our study to cases based on inpatient diagnoses only. Second, we conducted an E-value analysis for maternal-constant characteristics to assess how robust the observed associations are to unmeasured confounding. This test indicates how strongly an unmeasured confounder would need to be associated with both the exposure and the outcome to fully explain away the observed association.^26^ All statistical analyses were performed in STATA version 15 (StataCorp). This study was approved by Regional Ethical Vetting Board in Lund, Sweden (dnr: 2012/627).

## Results

**Figure 1.**
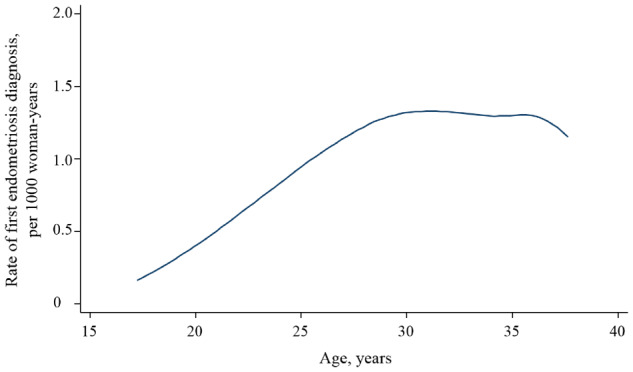
Incidence rate of first diagnosis of endometriosis in national inpatient and outpatient registers among Swedish women born in 1973–1987 and aged 15–40 during the follow-up period between 1987 and 2012.

Among 628 312 women, 8262 (1.31%) were diagnosed with endometriosis between the years 1987 and 2012. The incidence rate of endometriosis in women aged 15–40 years was 0.77 per 1000 woman-years. Incidence of endometriosis increased steadily from age 15, with a peak in incidence around age 30, and then gradually decreased until age 40 (Figure 1). The mean [standard deviation (SD)] age at first endometriosis diagnosis was 27.4 (5.0) years. In addition, we did not find any statistically significant difference in the incidence of endometriosis between women with complete case data and those without (*P* value = 0.5).

Table 1 shows index women’s maternal and birth characteristics. Almost half of our study population’s mothers had a secondary education, with an additional 32% having some tertiary education. A relatively small proportion of women (10%) were born to non-Swedish origin mothers, and half of those mothers came from other Nordic countries. The mean (SD) birthweight of index women was 3.4 (0.5) kg, whereas the mean (SD) for gestational age at delivery was 39.8 (1.8) weeks. Additionally 41% of the subjects were the first live-born child in the family. Maternal smoking during early pregnancy was prevalent in the subgroup of women with available information, and 29% of them were to some extent exposed to smoking *in utero*. This subgroup had similar maternal and birth characteristics compared with the total study population.

**Table 1.** Characteristics of women born in Sweden between 1973 and 1987

Characteristic	No.	%	No. of individuals with endometriosis	% of individuals with endometriosis
Maternal characteristics				
Mother’s highest educational level				
Elementary education	114 976	18.3	1766	21.4
Secondary education	308 119	49.0	4177	50.6
Shorter post-secondary (<3 years)	89 446	14.2	1054	12.8
University degree (≥3 years)	115 771	18.4	1265	15.3
Mother’s region of origin				
Sweden	568 992	90.6	7412	89.7
Other Nordic countries	30 708	4.9	470	5.7
North America	796	0.1	9	0.1
European countries except Nordic	11 887	1.9	165	2.0
Former Soviet states	4675	0.7	79	1.0
South America	1817	0.3	15	0.2
Middle Eastern	4870	0.8	67	0.8
Other Asia	3220	0.5	31	0.4
Africa	1347	0.2	14	0.2
Mother’s length of stay, years^a^				
≤29	4701	13.9	32	7.1
30–39	16 824	49.6	193	43.1
≥40	12 388	36.5	223	49.8
Mother’s age at child’s birth, years				
≤20	42 150	6.7	755	9.1
21–34	529 649	84.3	6899	83.4
≥35	56 513	9.0	618	7.5
Mother’s parity				
1	258 360	41.1	3501	42.4
2	240 060	38.2	3110	37.6
3	95 626	15.2	1222	14.8
≥4	34 266	5.5	429	5.2
Endometriosis in mother				
No	611 615	97.3	7776	94.1
Yes	16 697	2.7	486	5.9
Mother’s smoking in early pregnancy^b^				
Non-smoker	144 038	70.6	1039	63.9
Moderate smoker (1–9 cigarettes per day)	36 886	18.1	338	20.8
Heavy smoker (≥10 cigarettes per day)	22 967	11.3	249	15.3
Birth characteristics				
Birth year				
1973–1977	227 685	36.2	4159	50.3
1978–1982	201 792	32.1	2570	31.1
1983–1987	198 835	31.7	1533	18.6
Birth county				
Eastern Sweden	224 254	35.7	2842	34.4
Southern Sweden	274 295	43.7	3851	46.6
Northern Sweden	129 763	20.7	1569	19.0
Birthweight, kg				
<2.5	21 987	3.5	312	3.8
2.5–3.4	315 163	50.2	4365	52.8
3.5–4.4	278 878	44.4	3451	41.8
≥4.5	12 284	2.0	134	1.6
Gestational age, weeks				
Preterm birth ( <37)	24 523	3.9	286	3.5
Full term (37–41)	524 298	83.5	6833	82.7
Post term (≥42)	79 491	12.7	1143	13.8
Birthweight-for-gestational age				
Small-for-gestational age	6011	10.0	1046	12.7
Appropriate-for-gestational age	502 596	80.0	6521	78.9
Large-for-gestational age	62 705	10.0	695	8.4

a Available for 5% of study population born from non-Swedish born mothers.

b Available for 32% of study population born between 1982 and 1987.

## Maternal characteristics and endometriosis risk

### Mother’s education and region of birth

Mother’s educational level showed an inverse association with offspring’s incidence of endometriosis (Table 2). Specifically, the adjusted rate of endometriosis in women born to mothers with university education was 16% (95% CI, 9%–22%) lower than in those with mothers who only completed elementary education. After adjustment for other maternal characteristics, the incidence of endometriosis remained higher among women with mothers coming from other Nordic countries and the former Soviet states. The subset analysis on women with foreign-born mothers, however, indicated that the incidence of obtaining an endometriosis diagnosis from in-/out-patient care was not related to mother’s length of stay in Sweden (Table 2).

**Table 2.** Associations of maternal and birth characteristics with incidence of endometriosis, among women born in Sweden between 1973 and 1987

Characteristic	Woman-years at risk	No. of endometriosis cases	Minimally adjusted^a^	Further adjusted
			HR (95% CI)	HR (95% CI)
Maternal characteristic^b^				
Mother’s highest educational level				
Elementary education	2 080 833	1766	1.00	1.00
Secondary education	5 258 098	4177	0.96 (0.91–1.02)	0.96 (0.91–1.02)
Shorter post-secondary (<3 years)	1 487 068	1054	0.87 (0.80–0.94)	0.89 (0.82–0.96)
University degree (≥3 years)	1 903 633	1265	0.82 (0.76–0.88)	0.84 (0.78–0.91)
Mother’s region of origin				
Sweden	9 758 254	7412	1.00	1.00
Other Nordic countries	520 417	470	1.19 (1.09–1.31)	1.17 (1.06–1.28)
North America	11 786	9	1.08 (0.56–2.08)	1.14 (0.59–2.18)
European countries except Nordic	202 002	165	1.08 (0.92–1.26)	1.06 (0.91–1.24)
Former Soviet states	75 102	79	1.43 (1.14–1.78)	1.45 (1.16–1.81)
South America	25 789	15	0.84 (0.47–1.48)	0.83 (0.50–1.47)
Middle Eastern	71 422	67	1.34 (1.04–1.72)	1.22 (0.95–1.57)
Other Asia	46 168	31	0.97 (0.68–1.38)	0.94 (0.66–1.33)
Africa	18 692	14	1.10 (0.65–1.86)	1.08 (0.64–1.83)
Mother’s length of stay, years^c^				
≤29	43 280	32	1.00	1.00
30–39	258 474	193	0.68 (0.45–1.01)	0.68 (0.45–1.03)
≥40	227 503	223	0.82 (0.53–1.25)	0.84 (0.54–1.31)
Mother’s age at child’s birth, years				
≤20	769 484	755	1.00	1.00
21–34	9 048 798	6899	0.80 (0.74–0.86)	0.82 (0.76–0.89)
≥35	911 349	618	0.73 (0.65–0.81)	0.75 (0.67–0.85)
Mother’s parity				
1	4 461 520	3501	1.00	1.00
2	4 105 585	3110	0.97 (0.92–1.02)	1.00 (0.95–1.05)
3	1 596 496	1222	0.99 (0.93–1.06)	1.02 (0.96–1.10)
≥4	566 031	429	0.99 (0.89–1.09)	1.02 (0.92–1.14)
Endometriosis in mother	286 973	486	2.27 (2.07–2.49)	2.24 (2.04–2.46)
Mother’s smoking in early pregnancy^d^				
Non–smoker	1 779 419	1039	1.00	1.00
Moderate smoker (1–9 cigarettes per day)	457 706	338	1.26 (1.11–1.42)	1.18 (1.04–1.33)
Heavy smoker (≥10 cigarettes per day)	286 676	249	1.48 (1.28–1.69)	1.36 (1.18–1.57)
Birth characteristics^e^				
Birth county				
Eastern Sweden	3 834 327	2842	1.00	1.00
Southern Sweden	4 679 428	3851	1.11 (1.06–1.17)	1.10 (1.05–1.15)
Northern Sweden	2 215 877	1569	0.95 (0.90–1.02)	0.96 (0.90–1.02)
Birthweight, kg				
<2.5	375 071	312	1.14 (1.02–1.28)	1.16 (1.02–1.32)
2.5–3.4	5 404 474	4365	1.11 (1.06–1.16)	1.11 (1.06–1.16)
3.5–4.4	4 742 735	3451	1.00	1.00
≥4.5	207 351	134	0.89 (0.75–1.06)	0.89 (0.75–1.06)
Gestational age, weeks				
Preterm birth (<37)	400 143	286	0.94 (0.84–1.06)	0.92 (0.82–1.04)
Full term (37–41)	8 841 416	6833	1.00	1.00
Post term (≥42)	1 488 072	1143	0.95 (0.89–1.01)	0.95 (0.89–1.01)
Birthweight-for-gestational age (change per standard deviation)	10 729 631	8262	0.93 (0.91–0.95)	0.93 (0.91–0.95)

a Minimally adjusted models adjust for women’s birth year.

b Further adjusted models additionally adjust for other maternal characteristics except for mother’s smoking and length of stay.

c Further adjusted models additionally adjust for mother’s educational level, age at child’s birth, parity, and endometriosis in mother.

d Available for a subgroup of women born between 1982 and 1987 (*n* = 203 891). Further adjusted models additionally adjust for mother’s educational level, region of origin, age at child’s birth, parity, and endometriosis in mother.

e Further adjusted models additionally adjust for maternal characteristics except for mother’s smoking and length of stay, birthweight and birthweight-for-gestational-age, also adjust for gestational age.

### Mother’s age, parity and endometriosis

We found that women born to older mothers had a decreased incidence of endometriosis. (Table 2). However, such inverse association was not seen in the within-mother analysis, indicating that residual maternal factors confound the association and that this association should not be interpreted as a causal effect (Table 3). Mother’s parity showed no association with the incidence of endometriosis. As expected, endometriosis in the mother was a robust and strong predictor of higher incidence in the daughter (Table 2).

**Table 3.** Comparison of between-mother and within-mother associations for characteristics independently associated with endometriosis and varying between sisters, among women born in Sweden between 1973 and 1987

Characteristic	Mutually adjusted^a^
	HR (95% CI)
Mother’s age (change per decade)	
Between-mother^b^	0.82 (0.78–0.86)
Within–mother^c^	1.10 (0.95–1.27)
*P* value^d^	<0.00
Mother’s smoking in early pregnancy (any vs none)^e^	
Between-mother	1.34 (1.21–1.49)
Within-mother	1.36 (0.68–2.70)
*P* value	0.97
Birthweight (change per kg)^f^	
Between-mother	0.85 (0.81–0.89)
Within-mother	0.96 (0.85–1.09)
*P* value	0.06
Birthweight-for-gestational age (change per standard deviation)^f^	
Between-mother	0.92 (0.90–0.95)
Within-mother	0.98 (0.92–1.04)
*P* value	0.10
Small-for-gestational age (yes vs no)^f^	
Between-mother	1.21 (1.13–1.30)
Within-mother	1.12 (0.94–1.33)
*P* value	0.41

a Mutually adjusted models adjust for the between-mother and within-mother variables for the characterstic in question, and women’s birth year.

b Between-mother variables represent the average value of the characteristic in question across all of the mother’s offspring.

c Within-mother variables represent the departure of each woman from the family average (clustered in mother).

d *P* values are from Wald tests for equality of between-mother and within-mother coefficients.

e Available for a subgroup of women born between 1982 and 1987 (*n *= 203 891).

f Additionally adjusted for gestational age.

### Mother’s smoking

We found a strong positive association between maternal smoking in early pregnancy and incidence of early-onset endometriosis in daughters. This association persisted after accounting for mother’s educational level, region of origin, age and parity (Table 2). Our within-family comparison also suggested that the incidence of endometriosis increased among women whose mothers smoked during pregnancy. Although the within-mother coefficient was not significant, there was no evidence of heterogeneity when comparing the within- and between-associations, and the reduced sample size led to lower statistical power in this subset analysis for women with available maternal smoking information (Table 3).

### Birth characteristics and endometriosis risk

Lower birthweight was strongly associated with higher incidence of endometriosis in both minimally and further adjusted analyses (Table 2). There was no evidence that length of gestation was associated with endometriosis. For the standardized measure, birthweight-for-gestational age showed an inverse linear association with endometriosis, and remained unchanged after adjustment for maternal characteristics and gestational age (Table 2). There was not enough evidence to support that unmeasured maternal-level confounding underlies the association, because the between-mother and within-mother coefficients did not significantly differ from each other statistically (Table 3). The incidence of endometriosis was slightly increased for women born in Southern compared with Eastern Sweden (Table 2).

Mediation analyses indicated that an estimated 26% of the total association between maternal smoking and endometriosis in the daughter was mediated through birthweight-for-gestational age. Slow fetal growth, on the other hand, was not the reason for the associations of maternal education and history of endometriosis with daughter’s endometriosis (Table 4).

**Table 4.** Mediation of birthweight-for-gestational age on the associations for maternal characteristics associated with endometriosis, among women born in Sweden between 1973 and 1987

Characteristic	*P* value for exposure− mediator interaction	Adjusted HR (95% CI)^a^			
		Total association	Direct association	Indirect association	% Mediated by birthweight-for-gestational age
Mother’s highest educational level (high vs low)	0.27	0.90 (0.86–0.95)	0.91 (0.87–0.95)	1.00 (0.99–1.00)	5
Endometriosis in mother (yes vs no)	0.53	2.23 (2.04–2.43)	2.23 (2.05–2.44)	1.00 (0.99–1.00)	1
Mother’s smoking in early pregnancy (any vs none)^b^	0.02	1.24 (1.11–1.38)	1.17 (1.04–1.32)^c^	1.06 (1.02–1.09)	26

a Adjusted models adjust for maternal characteristics (i.e. mother’s educational level, region of birth, age at child’s birth, parity, endometriosis in mother) except the one under examined, gestational age, and women’s birth year.

b Available for a subgroup of women born between 1982 and 1987 (*n* = 203 891).

c Estimation based on birthweight-for-gestational age at control value 0.

### Sensitivity analyses

Our main findings were very similar in the sensitivity analyses that restricted endometriosis cases to inpatient diagnoses (results not shown).The E-value analysis implies that conditional on all measured maternal characteristics, an unmeasured confounder needs to be strongly associated with both mother’s and daughter’s endometriosis to fully explain away the association, but weaker confounding could not. On the other hand, relatively less unmeasured confounding would suffice to explain away the associations of maternal education, region of origin, and women’s birth county with the incidence of endometriosis (Supplementary Table 1, available as Supplementary data at *IJE* online). 

## Discussion

In this total-population based cohort of 628 312 women, we found that maternal smoking during pregnancy, lower birthweight and lower birthweight-for-gestational age were all associated with an increase in the incidence of endometriosis during the early to mid-reproductive period. All these perinatal characteristics showed clear dose−response relationships with subsequent endometriosis risk, and were confirmed as robust predictors through extensive confounding adjustments and within-family comparisons. We estimated a 26% reduction in the total association between maternal smoking and risk of early-onset of endometriosis through birthweight-for-gestational age. We also observed associations of maternal history of endometriosis, lower education and region of origin with risk of endometriosis in the daughter.

### Comparison with previous research

The main finding our study is a positive association between maternal smoking and endometriosis in daughters. Earlier findings on the effect of smoking on risk of endometriosis were inconclusive and conflicting across the life-course periods in which the woman was exposed to smoking. Adult smoking was generally considered as a protective factor for endometriosis.^27^^,^^28^ Considering that endometriosis is an oestrogen-dependent condition, it is possible that the reduction/deficiency in oestrogen levels among smokers could explain the inverse association. Intriguingly, in the present study, we observed a dose−response association between *in utero* exposure to smoking and endometriosis in the opposite direction. This finding is contradictory of some previous studies that were based on small samples (range, 32–310 cases) and used a retrospective design.^9^^,^^13^^,^^14^^,^^18^

To our knowledge, this study is the first population-based study with prospectively collected data during pregnancy to examine maternal smoking and endometriosis. It should, however, be noted that our findings may only be applicable to early-onset endometriosis, since the available information in the register only allowed us to follow women until ages 25–30 years in the subgroup of women with smoking data available. We hypothesize that the specific mechanism behind the risk effect of exposure to smoking *in utero* is different from adult smoking (i.e. the antioestrogenic effect of direct smoking^29^). It may be that fetal exposure to dioxins or other toxic components from cigarettes alter the immune function of the fetuses, which in turn makes them more vulnerable to inflammation conditions and results in ectopic endometrium.^10^ Indeed, both animal and human studies have suggested that environmental exposure to dioxin could increase the risk of endometriosis potentially through impaired immune defence.^30^^,^^31^ In line with our finding, a French study found that exposure to passive smoking during childhood was also related to endometriosis.^32^ Apart from that, we observed that a quarter of the adverse effect from maternal smoking on endometriosis may be attributable to the slow growth rate in fetal life.

The inverse association between maternal education and endometriosis has not been reported and is somewhat unexpected given previous evidence from North America showing that the risk of endometriosis was higher among women with higher adult socio-economic positions.^11^^,^^33^ These inconsistent findings might partly arise from the egalitarian health care system in Sweden, which ensures good access to health care irrespective of socio-economic conditions. In the Swedish context low-educated women have better access to health care, which should result in a higher number of diagnosed cases of endometriosis, thereby evening out the incidence rates between the groups. In the US for example, poorer access to health care at lower socio-economic statuses may result in a higher share of undiagnosed cases, which leads to higher (observed) incidence among those with greater resources.

Moreover, we found that women born to foreign-born mothers, especially those who came from other Nordic countries and the former Soviet states had higher risks of endometriosis. This points in the same direction as a previous Swedish study in which the associations with women’s own country of birth was examined.^34^ Regarding the excess risk among women born to mothers who came from former Soviet states, we speculate that such associations might reflect the potential psychosocial stress perceived by women during their childhood, which has been linked to the aetiology of endometriosis.^35^ Interestingly, this hypothesis would also be relevant for interpreting maternal smoking as an indicator of maternal stress and a less optimal psychosocial environment for their children. This argument does not hold for the Nordic mothers, however, pointing to the need for more research regarding these women.

A previous study has identified an inverse association between maternal age at child’s birth and the risk of endometriosis in daughters.^36^ By contrast, our within-family comparison suggested that such association was spurious and entirely the product of shared maternal genetic or environmental factors. On the other hand, in line with many previous findings and the heritability feature of endometriosis,^37–39^ we found that the mother’s history of endometriosis was a robust predictor for daughter’s endometriosis.

We confirm that lower birthweight and lower birthweight-for-gestational age were robustly associated with an elevated risk of endometriosis. Our finding is consistent but improved upon many previous studies,^15^^,^^16^^,^^40^ suggesting that the risk effect of low birthweight was related to slow fetal growth rate rather than preterm birth. These findings are in line with the developmental origins of health and disease theory, postulating that exposure to environmental insults during the critical period could exert a long-term impact on the structure and metabolism of the human body.^1^^,^^2^ Intrauterine growth restriction can be seen as an indicator for a range of adverse conditions, such as disruptions in early endocrine status, poor reproductive function, and subsequent alterations in hormone secretion, all of which result in adult susceptibility to endometriosis.^2^ Additionally, low birthweight has been suggested to have an impact on immune function in later life,^41^ which might make some women more vulnerable to develop inflammatory conditions, like endometriosis.

### Strengths and limitations

To our knowledge, this is the first study based on total-population design and prospectively collected data to investigate perinatal risk factors for endometriosis. Our within-family analyses allowed us to control for unmeasured maternal-level confounding. The high diagnostic accuracy in the inpatient data and validity of the national registers minimize potential biases from disease misclassification and recall bias.^20^ Our findings should, however, be interpreted with consideration of several limitations. One limitation is that we only captured cases recorded in the inpatient and outpatient care, and we may miss cases in primary care. Thus, our findings may not generalize to less severe and undiagnosed cases. Another limitation is the length of follow-up, our women were aged from 25 to 40 years at the end of follow-up. Although incidence of endometriosis reaches the peak at around age 30,^8^ we still cannot capture those cases presenting in later reproductive ages, which somewhat confines the generalizability of our study. A third limitation is that self-reported information on maternal smoking during pregnancy was only collected at the first antenatal visit. This mainly reflects the fetus’ exposure to smoking during the first trimester, although a previous validation study has shown that this smoking measurement agreed well with the women’s levels of cotinine at delivery.^42^ In addition, considering the correlation between maternal smoking during pregnancy and the postnatal period, we cannot rule out the potential adverse effect from passive smoking during childhood. Identifying the sensitive period for passive smoking on endometriosis risk should be the priority for future research. Finally, our within-family comparison cannot account for unmeasured family-varying confounding (e.g. individual lifestyle factors) and might have lacked statistical power.

## Conclusion

The present study finds support for fetal origins of endometriosis. Exposure to maternal smoking and growth restriction during the fetal period in particular were associated with an increased risk of endometriosis at early to mid-reproductive life. We hope our findings will help in understanding the cause of endometriosis and identifying targets for public health intervention.

## Funding

This study was supported by grants from the European Union’s Horizon 2020 research and innovation programme under grant agreement No 635316 (ATHLOS project), the Swedish Research Council (Projects No 2013–5104 and 2013–5474), and the China Scholarship Council (No 201600160078) (M.G.).

## Supplementary Material

dyz140_Supplementary_Data
