# Corrigendum to: Associations of perinatal characteristics with endometriosis: a nationwide birth cohort study

**DOI:** 10.1093/ije/dyz225

**Published:** 2019-10-31

**Authors:** Menghan Gao, Kirk Scott, Ilona Koupil

First published online: 3 July 2019, *Int J Epidemiol* 2020;49:537–47. doi: https://doi.org/10.1093/ije/dyz140

In the footnotes of Tables 1-4, the range of birth years in the subgroup of women with mother’s smoking information available was incorrectly stated as between 1982 and 2012. The correct birth year range is between 1982 and 1987. In the Discussion, the age at follow-up for the subgroup of women with mother’s smoking data available was incorrectly stated as 25–34 years. The correct age range is 25–30 years and the respective statement should read: “… the available information in the register only allowed us to follow women until ages 25–30 years in the subgroup of women with smoking data available”.

The article has been corrected.

