# Supplementary material for: Associations of perinatal characteristics with endometriosis: a nationwide birth cohort study
Source: Int J Epidemiol. 2019 Jul 3;49(2):537–47. doi: 10.1093/ije/dyz140 (PMC7266550; doi:10.1093/ije/dyz140)
Supplement: dyz140_Supplementary_Data [file dyz140_supplementary_data.docx]

**Supplementary Table 1.** E-values of associations for maternal characteristics independently associated with endometriosis and do not vary between sisters, among women born in Sweden between 1973 and 1987

| **E-values ^a^** | **Estimate ^b^** | **Confidence Interval ^c^** |
| --- | --- | --- |
|  | **Hazard Ratio** | **Hazard Ratio** |
| Mother’s highest educational level |  |  |
| Elementary education | 1.00 (Reference) | 1.00 (Reference) |
| Shorter post-secondary (<3 years) | 1.50 | 1.25 |
| University degree (≥3 years) | 1.67 | 1.43 |
| Mother’s region of origin |  |  |
| Sweden | 1.00 (Reference) | 1.00 (Reference) |
| Other Nordic countries | 1.62 | 1.31 |
| Former Soviet states | 2.26 | 1.59 |
| Endometriosis in mother | 3.91 | 3.50 |
| Birth county |  |  |
| Eastern Sweden | 1.00 (Reference) | 1.00 (Reference) |
| Southern Sweden | 1.43 | 1.28 |

^a^ Only values showed statistically significant associations with endometriosis in Table 2 were further examined here.

^b^ The minimum strength of associations that an unmeasured confounder need to have with both the maternal characteristic of interest and endometriosis to fully explain away the respective estimate, after adjustment for other measured maternal characteristics.

^c^ The minimum strength of associations that an unmeasured confounder need to have with both the maternal characteristic of interest and endometriosis to move the confidence interval to include the null, after adjustment for other measured maternal characteristics.
